# Treatment Patterns for Patients With Unresected Stage III NSCLC: Analysis of the Surveillance, Epidemiology, and End Results (SEER) Database

**DOI:** 10.3389/fonc.2022.874022

**Published:** 2022-06-17

**Authors:** Shijie Shang, Ruiyang Wang, Fei Wang, Meng Wu, Dawei Chen, Jinming Yu

**Affiliations:** ^1^ Department of Radiation Oncology, Shandong University Cancer Center, Jinan, China; ^2^ Department of Radiation Oncology and Shandong Provincial Key Laboratory of Radiation Oncology, Shandong Cancer Hospital and Institute, Shandong First Medical University and Shandong Academy of Medical Sciences, Jinan, China; ^3^ Research Unit of Radiation Oncology, Chinese Academy of Medical Sciences, Jinan, China

**Keywords:** non-small cell lung cancer (NSCLC), stage III, unresected, radiotherapy, SEER database

## Abstract

**Background:**

Recently, immunotherapy (IO) has shown striking survival improvement in unresectable stage III non-small cell lung cancer (NSCLC). However, the role of chemo-radiotherapy (CRT) for improvement in outcomes should not be disregarded. This study aimed to compare the treatment patterns and illustrate the impact of radiotherapy on the cancer-specific survival (CSS) and overall survival (OS) of patients with unresected locally advanced stage III NSCLC.

**Methods:**

We retrospectively analyzed the data of patients with stage III NSCLC patients who did not undergo surgery from the National Cancer Institute Surveillance, Epidemiology, and End Results (SEER) database between 2001 and 2016, and three continuous years were regarded as one unit. Using the Kaplan-Meier method, we identified the CSS and OS. Then, a linear regression model was graphed to analyze the correlation between median survival of CSS or OS and calendar years in the radiotherapy alone, chemotherapy alone, and CRT groups.

**Results:**

A total of 20986 patients were included in this study. In the overall cohort, CSS and OS improved consistently. To explore the reason for the improved survival, patients were divided into three different cohorts: radiotherapy alone, chemotherapy alone, and CRT. From 2001 to 2015, the median CSS improved persistently, 7, 8, 8, 9, and 11 months in the radiotherapy alone group and 12, 13, 15, 17, 19 months in the CRT group, but improvement in outcomes was less consistent in the chemotherapy alone group (10, 9, 11, 12, 12 months). To better visualize the correlation between CSS and calendar year, linear regression was performed, yielding r^2^ = 0.8032, P = 0.0395 for the radiotherapy alone group; r^2^ = 0.7206, P = 0.0689 for the chemotherapy alone group; and r^2^ = 0.9878, P = 0.0006 for the CRT group. Similar findings were observed in the OS data. In addition to this, we also analyzed different pathological types and also obtained the same results.

**Conclusions:**

The survival of patients with unresectable stage III NSCLC has improved substantially, and the most pronounced and consistent improvements were observed in the CRT group. In addition to IO, radiotherapy played an essential role in the treatment of unresectable stage III NSCLC in the past years and should be considered in the design of clinical trials.

## Introduction

Non-small cell lung cancer (NSCLC) accounts for approximately 85% of lung cancers and is a major cause of cancer-related deaths worldwide (1). Approximately 30% of patients are diagnosed with stage III disease, which is often unresectable. Stage III NSCLC is also called “locally or locoregionally advanced disease.” It is highly heterogeneous, with a wide spectrum of disease distribution, and requires a multidisciplinary treatment approach (2).

In the last few decades, there has been modest progress in the treatment modalities for unresectable stage III NSCLC, from definitive radiotherapy alone to sequential and concurrent chemo-radiotherapy (CRT). The treatment goal for patients with unresectable, locally advanced stage III NSCLC is mainly curative to prevent local recurrence and reduce the occurrence of distant metastases. Concurrent CRT is currently the standard treatment regimen for patients with unresectable stage III NSCLC (3–7).

Additionally, radiotherapy is a critical component of the definitive treatment regimen for locally advanced NSCLC. Its role in this field has been summarized in several reviews (8, 9). Several studies have evaluated the survival of patients with unresectable stage III NSCLC undergoing radiotherapy alone by comparing different radiotherapy fractionation and dosing intensities (10–12). Existing data demonstrate improvement in symptoms following radiation treatment (10, 13–15), but the mortality benefits are less clear. Furthermore, there are no randomized controlled trials comparing radiotherapy alone to placebo in the treatment of unresectable stage III NSCLC.

This study aimed to compare the treatment patterns and illustrate the impact of radiotherapy on the cancer-specific survival (CSS) and overall survival (OS) of patients with unresected locally advanced stage III NSCLC. To do so, we retrospectively analyzed the CSS and OS data from patients enrolled in the Surveillance, Epidemiology, and End Results (SEER) cancer database.

## Materials and Methods

### Study Population

**Figure 1:**
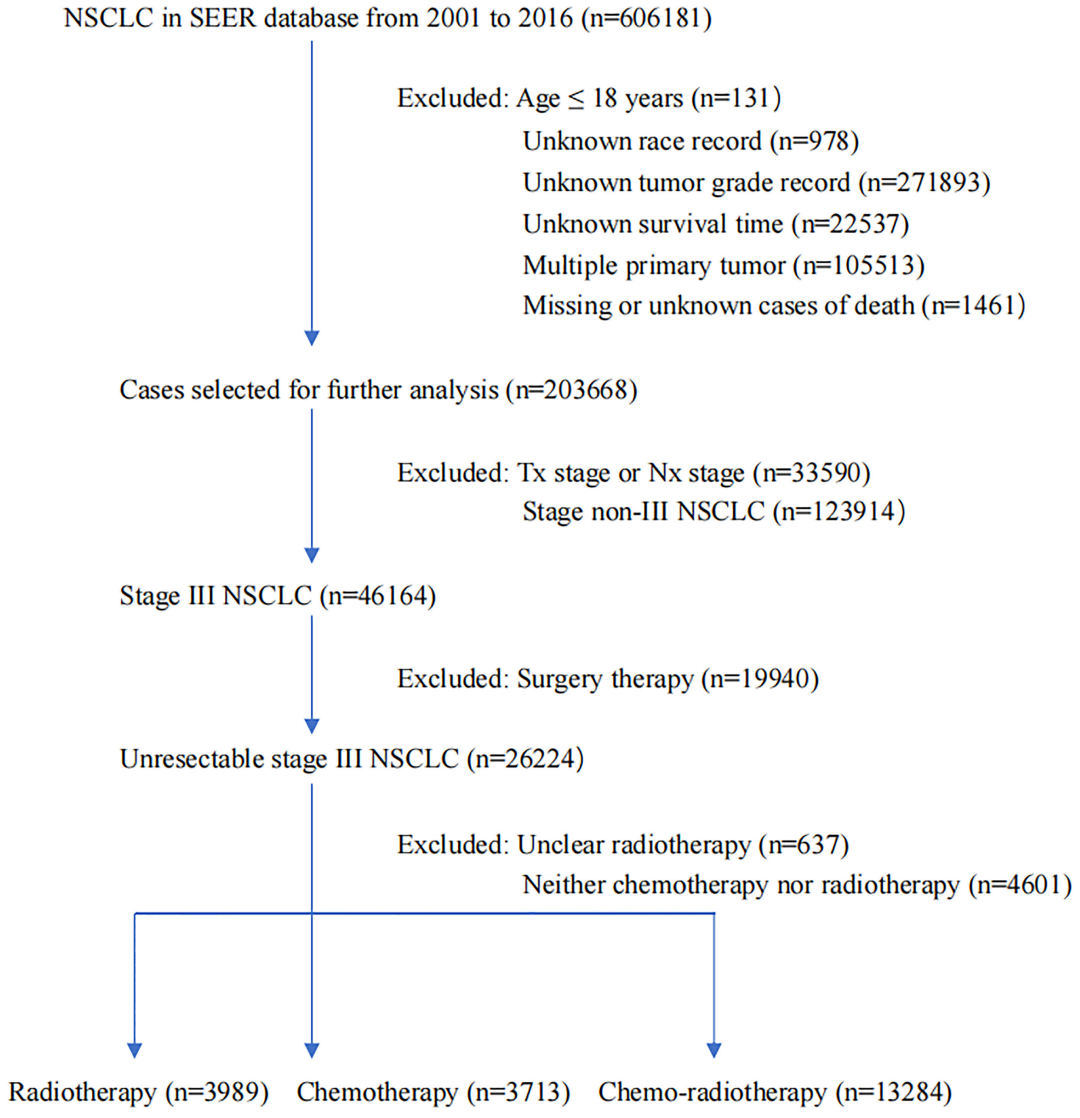
The flowchart of study population selection.

The SEER database integrates data from different population-based cancer cases, covering approximately 34% of the United States population. Our analysis data consisted of 20986 unresected patients with histologically confirmed stage III NSCLC diagnosed between 2001–2016. Three continuous years were regarded as one unit. Patients included in the study had to meet the following criteria: specific age information, definite race recorded, accurate pathology typing, no distant metastasis, clear therapy data, active follow-up, and the presence of only one primary tumor. Those with incomplete staging, unknown tumor grade, unknown survival time, missing or unknown cause of death, and who received surgical treatment were excluded (**Figure 1**). The enrolled patients received radiotherapy alone, chemotherapy alone, or CRT. It was not detailed in the SEER database whether chemotherapy was concurrent with or sequential to radiotherapy. Patients with insufficient data on whether they underwent radiotherapy or refused were also excluded. The primary analysis endpoint was CSS, measured from initial diagnosis to death from lung cancer, and OS, measured from initial diagnosis to death from any cause or censoring. The cause of death provided in the SEER database originated from the National Center for Health Statistics’ database of consolidated death certificates from the Vital Statistics Office in each state.

### Statistical Analysis

For all cases, the following variables were analyzed: age, race, sex, pathological grade, American Joint Committee on Cancer stage, and therapy regimen. Differences in the distribution of the baseline characteristics were summarized. Univariate and multivariate Cox proportional hazards regression models were used to evaluate the risk of mortality and conduct a subgroup analysis. The Kaplan Meier method was used to estimate the CSS and OS of the patients in the three treatment groups. Survival curves were graphed using the median CSS and OS calculated for each group. The median CSS and OS among the three treatment groups were also compared using linear regression. We used SPSS Statistics 24 (IBM, Armonk, New York, United States) and GraphPad Prism 9 (GraphPad Software, Inc. San Diego, California, United States) for all statistical analyses. Statistical significance was set at *P < 0.05.*

## Results

Using the SEER database, a total of 20986 patients with unresected stage III NSCLC were included in this retrospective study; all of whom underwent radiotherapy alone, chemotherapy alone, or CRT. The demographic characteristics of the patients are summarized in **Table 1**. Patients were divided into three cohorts: radiotherapy alone, chemotherapy alone, and CRT. There were notable differences in age, race, pathological grade, T stage, and N stage of the groups. Specifically, patients treated with the three treatment modalities were older and had higher pathological grades. The white race also makes up the majority of the three treatment modalities. Among the three different treatment modalities, patients with T4 stage or N2 stage accounted for the largest proportion. The sex distribution of patients who underwent radiotherapy alone, chemotherapy alone, or CRT was not significantly different.

**Table 1:** Baseline characteristics of patients with unresected stage III non-small cell lung cancer.

Variables	Total (%)	Radiotherapy (%)	Chemotherapy (%)	Chemo-radiotherapy (%)
Total	20986	3989	3713	13284
Age				
<65	8389 (40.0)	915 (22.9)	1346 (36.3)	6128 (46.1)
≥65	12597 (60.0)	3074 (77.1)	2367 (63.7)	7156 (53.9)
Race				
White	16600 (79.1)	3114 (78.1)	2868 (77.2)	10618 (79.9)
Black	2986 (14.2)	643 (16.1)	531 (14.3)	1812 (13.7)
Other	1400 (6.7)	232 (5.8)	314(8.5)	854 (6.4)
Sex				
Male	12452 (59.3)	2320 (58.2)	2088 (56.2)	8044 (60.6)
Female	8534 (40.7)	1669 (41.8)	1625 (43.8)	5240 (39.4)
Grade				
I-II	7105 (33.9)	1464 (36.7)	1273 (34.3)	4368 (32.9)
III-IV	13881 (66.1)	2525 (63.3)	2440 (65.7)	8916 (67.1)
T stage				
T1	1633 (7.8)	259 (6.5)	257 (6.9)	1117 (8.4)
T2	5430 (25.9)	920 (23.1)	801 (21.6)	3709 (27.9)
T3	3740 (17.8)	695 (17.4)	493 (13.3)	2552 (19.2)
T4	12743 (48.5)	2115 (53.0)	2162 (58.2)	5906 (44.5)
N stage				
N0	2953 (14.1)	755 (18.9)	657 (17.7)	1541 (11.6)
N1	1199 (5.7)	247 (6.2)	200 (5.3)	752 (5.7)
N2	13262 (63.2)	2501 (62.7)	2189 (59.0)	8572 (64.5)
N3	3572 (17.0)	486 (12.2)	667 (18.0)	2419 (18.2)

**Figure 2:**
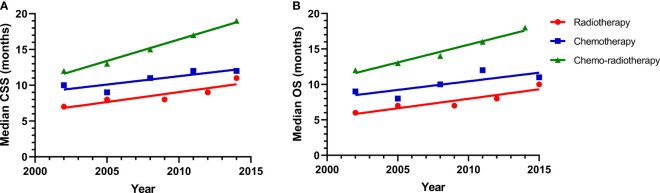
**(A)** A linear regression model for median survival of CSS in the radiotherapy alone (r = 0.8032, P = 0.0395), chemotherapy alone (r = 0.7206, P = 0.0689), and chemo-radiotherapy groups (r = 0.9878, P = 0.0006); **(B)** A linear regression model for median survival of OS in the radiotherapy alone (r = 0.8487, P = 0.0262), chemotherapy alone (r = 0.6080, P = 0.1199), and chemo-radiotherapy groups (r = 0.9868, P = 0.0022).

Kaplan-Meier survival curves of CSS and OS for the three treatment modalities are shown in **Supplementary Figure**. Based on Kaplan-Meier survival curves, we know that patients benefit the most from CRT. We explored the greatest contribution of radiotherapy or chemotherapy to patient survival. The median CSS curves of patients who received radiotherapy alone, chemotherapy alone, or CRT are shown in **Figure 2**. From 2001 to 2015, the median CSS was 7, 8, 8, 9, 11 months for patients treated with radiotherapy alone; 10, 9, 11, 12, 12 months for patients who received chemotherapy alone; and 12, 13, 15, 17, 19 months for the CRT group. To better illustrate the correlation between median CSS and calendar year, linear regression was performed, yielding r^2^ = 0.8032, P = 0.0395 for the radiotherapy alone group; r^2^ = 0.7206, P = 0.0689 for the chemotherapy alone group; and r^2^ = 0.9878, P = 0.0006 for the CRT group. The median OS curves for the three cohorts are also shown in **Figure 2**. The median OS for patients receiving radiotherapy alone was 6, 7, 7, 8, 10 months, compared to 9, 8, 10, 12, 11 months in the chemotherapy alone group and 12, 13, 14, 16, 18 months in the CRT group. Linear regression was also used to describe the correlation between median OS and calendar year, yielding r^2^ = 0.8487, P = 0.0262 for the radiotherapy alone group; r^2^ = 0.6080, P = 0.1199 for the chemotherapy alone group; and r^2^ = 0.9868, P = 0.0022 for the CRT group. Kaplan-Meier analyses showed improved CSS and OS in the radiotherapy alone cohort. The CSS and OS rates of patients who were treated with radiotherapy alone were significantly better than those of patients who received chemotherapy alone.

**Figure 3:**
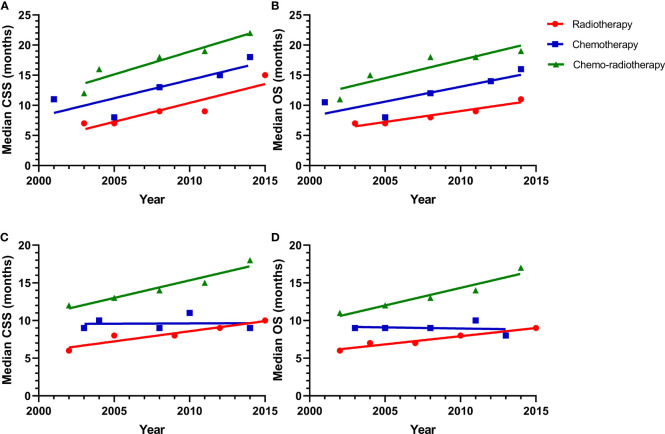
**(A)** A linear regression model for median survival of CSS of patients with adenocarcinoma in the radiotherapy alone (r = 0.8304, P = 0.0313), chemotherapy alone (r = 0.7036, P = 0.0758), and chemo-radiotherapy groups (r = 0.8900, P = 0.0160); **(B)** A linear regression model for median survival of OS of patients with adenocarcinoma in the radiotherapy alone (r = 0.9268, P = 0.0086), chemotherapy alone (r = 0.7069, P = 0.0744), and chemo-radiotherapy groups (r = 0.8176, P = 0.0351); **(C)** A linear regression model for median survival of CSS of patients with non-adenocarcinoma in the radiotherapy alone (r = 0.8995, P = 0.0140), chemotherapy alone (r = 0.001392, P = 0.9525), and chemo-radiotherapy groups (r = 0.9245, P = 0090); **(D)** A linear regression model for median survival of OS of patients with non-adenocarcinoma in the radiotherapy alone (r = 0.9503, P = 0.0048), chemotherapy alone (r = 0.02941, P = 0.7827), and chemo-radiotherapy groups (r = 0.9245, P = 0.0090).

In addition, we analyzed the survival of patients with different pathological types. All pathological types were divided into adenocarcinoma (AC) and non-AC. The analyses of the median CSS and OS for the patients with lung AC was shown in **Figure 3**. The median CSS was 7, 7, 9, 9, 15 months for patients with lung AC in the radiotherapy alone cohort. 11, 8, 13, 15, 18 months for patients received chemotherapy alone; and 12, 16, 18, 19, 22 for the CRT group. Linear regression was also performed to present the correlation between median CSS and calendar year, yielding r^2^ = 0.8304, P = 0.0313 for the radiotherapy alone group; r^2^ = 0.7036, P = 0.0758 for the chemotherapy alone group; and r^2^ = 0.8900, P = 0.0160 for the CRT group. The median OS was 7, 7, 8, 9, 11 months in the radiotherapy alone group, while it was 10.5, 8, 12, 14, 16 months in the chemotherapy alone group and 11, 15, 18, 18, 19 for the CRT cohort. Linear regression was analyzed to yield r^2^ = 0.9268, P = 0.0086 for the radiotherapy alone group; r^2^ = 0.7069, P = 0.0744 for the chemotherapy alone group; and r^2^ = 0.8176, P = 0.0351 for the CRT group. The similar analysis was applied to non-AC, which was also shown in **Figure 3**. The median CSS was 6, 8, 8, 9, 10 months for patients who received radiotherapy alone; 9, 10, 9, 11, 9 months for them treated with chemotherapy alone; and 12, 13, 14, 15, 18 months in the CRT cohort. Linear regression was used to delineated the correlation between median CSS and calendar year, yielding r^2^ = 0.8995, P = 0.0140 for the radiotherapy alone group; r^2^ = 0.00132, P = 0.9525 for the chemotherapy alone group; and r^2^ = 0.9245, P = 0.0090 for the CRT group. Similar result was found in the OS data. The median OS was 6, 7, 7, 8, 9 months in the radiotherapy alone group compared to 9, 9, 9, 10, 8 months in the chemotherapy alone group and 11, 15, 18, 18, 19 for the CRT group. Linear regression was applied to yield r^2^ = 0.9503, P = 0.0048 for the radiotherapy alone group; r^2^ = 0.02941, P = 0.7827 for the chemotherapy alone group; and r^2^ = 0.9245, P = 0.0090 for the CRT group.

In the Cox hazard analysis, the results of variables according to CSS and OS on the univariate analysis, including age, race, sex, pathological grade, TN stage, and therapy regimen, are shown in **Table 2**. The outcomes of the multivariate analysis are presented in **Table 3**. According to the results of the univariate Cox hazard regression model, the non-white race and female sex were associated with longer CSS and OS, while increasing age, high pathologic grade, and higher T stage were associated with shorter CSS and OS. In the multivariate analysis of CSS and OS, age, race, sex, pathological grade, and T stage were statistically significant (P < 0.05). Multivariate analysis shown that increasing age, high pathologic grade, and higher T stage were independent risk factors for poor prognosis. The non-white race and female sex were independent predictors of prolonged survival. These factors may potentially influence patient’s treatment options.

**Table 2:** Univariate and multivariate analysis for CSS.

Variables	Univariate analysis			Multivariate analysis		
	HR	95% CI	P value	HR	95% CI	P value
Age	1.141	1.106-1.177	0.001			
<65				Reference		
≥65				1.077	1.042-1.112	0.001
Race	0.946	0.921-0.972	0.001			
White				Reference		
Black				0.948	0.907-0.991	0.019
Other				0.884	0.830-0.942	0.001
Sex	0.844	0.818-0.871	0.001			
Male				Reference		
Female				0.837	0.811-0.864	0.001
Grade	1.09	1.055-1.126	0.001			
I-II				Reference		
III-IV				1.138	1.101-1.176	0.001
T stage	1.154	1.136-1.171	0.001			
T1				Reference		
T2				1.126	1.188-1.356	0.001
T3				1.338	1.248-1.435	0.001
T4				1.577	1.481-1.679	0.001
N stage	1.004	0.986-1.022	0.665	Not entered		
N0						
N1						
N2						
N3						
Therapy	0.806	0.791-0.821	0.001			
Chemotherapy				Reference		
Radiotherapy				1.208	1.150-1.270	0.001
Chemo-radiotherapy				0.706	0.677-0.735	0.001

**Table 3:** Univariate and multivariate analysis for OS.

Variables	Univariate analysis			Multivariate analysis		
	HR	95% CI	P value	HR	95% CI	P value
Age	1.198	1.162-1.234	0.001			
<65				Reference		
≥65				1.123	1.089-1.159	0.001
Race	0.933	0.909-0.958	0.001			
White				Reference		
Black				0.945	0.905-0.986	0.009
Other				0.862	0.812-0.916	0.001
Sex	0.844	0.819-0.870	0.001			
Male				Reference		
Female				0.837	0.812-0.863	0.001
Grade	1.067	1.034-1.100	0.001			
I-II				Reference		
III-IV				1.122	1.087-1.159	0.001
T stage	1.133	1.117-1.149	0.001			
T1				Reference		
T2				1.233	1.159-1.312	0.001
T3				1.269	1.188-1.355	0.001
T4				1.485	1.401-1.575	0.001
N stage	1.002	0.985-1.018	0.857	No entered		
N0						
N1						
N2						
N3						
Therapy	0.81	0.796-0.825	0.001			
Chemotherapy				Reference		
Radiotherapy				1.248	1.190-1.309	0.001
Chemo-radiotherapy				0.718	0.690-0.747	0.001

## Discussion

This retrospective study was conducted using data from the SEER database, which revealed that approximately 20986 patients with stage III NSCLC were not resected due to medical or surgical reasons. Although the efficacy of all the three treatment patterns, which included radiotherapy alone, chemotherapy alone and CRT, improved modestly, the most obvious improvement was found in the CRT group. The potential reason might be that the drugs of chemotherapy, such as cisplatin and paclitaxel, could synergize the effect of radiotherapy (16).

Although CRT is the most beneficial, radiotherapy was associated with a statistically significant improvement in CSS and OS for patients with unresected stage III NSCLC, consistent with findings from clinical practice, which explains the importance of radiotherapy in the treatment of unresected stage III NSCLC. For the analysis of different pathological types, the importance of radiotherapy has also been shown. The reasons underlying these improvements may lie in advances in radiotherapy technology (17, 18) and indications of concurrent CRT as the standard therapy (3, 19). Radiotherapy has changed from two-dimensional traditional radiotherapy to three-dimensional conformal radiotherapy, to intensity-modulated radiotherapy, to image-guided radiotherapy, to proton radiotherapy, which is the technical development process of radiotherapy technology gradually moving towards precision radiotherapy. Patients are increasingly benefiting from radiation therapy due to continuous advancements in radiotherapy techniques. Non-randomized studies have shown that some advanced radiotherapy techniques, such as 4D-CT four-dimensional computed tomography or positron emission tomography-computed tomography simulation techniques, combined with image-guided radiotherapy, tomotherapy, and proton radiotherapy, can reduce radiotherapy toxicity and improve efficacy, compared with the conventional radiation therapy. At the same time, radiotherapy modalities are also continuously optimized. For example, relevant clinical studies of hyperfractionated or accelerated hyperfractionated radiotherapy have shown that reducing the overall treatment time can significantly improve long-term survival (20, 21). Another reason for the improvement is related to the application of immunotherapy (22–24) and other treatments (10, 25, 26). In multivariate analyses of CSS and OS, some factors, such as age, race, sex, pathological grade, and T stage, may potentially influence patient treatment choices, but less so than treatment mode.

As to the different histology type, especially for AC and non-AC, the efficacy of both of the radiotherapy alone or the CRT improved significantly, and only the differentia was shown in the chemotherapy alone group. For patients with AC, the efficacy of the chemotherapy alone improved significantly, whereas this is not the case for non-AC. So, it might be speculated that patients with AC might be more related with the improvement of radiotherapy techniques and the update of chemotherapy regimens in recent years, however, for patients with non-AC, it might be only related with the improvement of radiotherapy techniques alone.

Though survival has improved over time, the improvements have been modest, which suggests that the prevention of local and distant failure remains a great challenge. Although our study period did not cover the introduction of durvalumab after CRT for patients with unresectable locally advanced stage III NSCLC, we acknowledge that the PACIFIC regimen has led to improvements in OS and progression-free survival (PFS) (27, 28), helping address the urgent needs of these patients.

Our study has several strengths and limitations. One strength is the use of large, nationally representative data with long-term follow-up. The patients included in the treatment group represented a diverse group of NSCLC patients, as opposed to many smaller trials from single institutions. Therefore, we anticipate that the generalizability of our results will be meaningful.

The limitations of this study are as follows. First, because of the use of observational data in our study, the assignment of patients in the three treatment groups was not random. This treatment allocation process could have generated systematic errors in the distribution of baseline characteristics among patients who received radiotherapy alone, chemotherapy alone, or CRT. Second, there is a lack of information about patients’ performance status and comorbidities in the SEER database, which are major prognostic factors and the leading reason for patients’ treatment choices. In addition, the SEER database does not detail if the treatment was completed and if chemotherapy was concurrent or sequential to radiotherapy. The lack of information regarding the mode of radiation treatment is another limitation of our study since the radiation dose, fractionation schedule, and radiotherapy techniques are all important factors in NSCLC. We were unable to distinguish between three-dimensional techniques, intensity-modulated radiotherapy, and stereotactic body radiotherapy. Many advanced radiotherapy techniques, e.g. image-guided radiotherapy and SBRT, were introduced more recently, so that patients treated in earlier years can be assumed to not have received radiotherapy with these advances techniques.

No randomized controlled trials have evaluated the effectiveness of radiotherapy compared to the placebo. Thus, the results of our study should help clinicians guide patients with stage III NSCLC who are not candidates for surgery and inform them of the advantages of radiotherapy in routine clinical practice.

In summary, our study suggests that radiotherapy is associated with improved survival in patients with unresected stage III NSCLC. The observed increase in survival was modest; however, this improvement in survival was comparable to that achieved with the use of chemotherapy for unresected stage III NSCLC. When designing clinical trials, we should also fully consider the improvement of patient survival following advances in radiotherapy. Changes in the current radiation technology or new therapeutic strategies are necessary to improve the outcomes of patients with unresected stage III NSCLC. Further prospective studies should aim to confirm these results and better define the optimal radiotherapy regimen in this population.

## Data Availability Statement

The original contributions presented in the study are included in the article/**Supplementary Material**. Further inquiries can be directed to the corresponding authors.

## Author Contributions

DC had access to all the data in the study and takes responsibility for the integrity of the data and the accuracy of the data analysis. Concept and design: SS, RW, DC, JY. Acquisition, analysis, or interpretation of data: SS, RW, DC. Drafting of the manuscript: SS, RW, DC. Critical revision of the manuscript for important intellectual content: SS, RW, FW, MW, DC, JY. Statistical analysis: SS, RW, DC. Obtained funding: DC, JY. All authors contributed to the article and approved the submitted version.

## Funding 

DC has received grants from National Natural Science Foundation of China (82172676), Science Foundation of Shandong (ZR2021YQ52, ZR2020LZL016), Foundation of Bethune Charitable Foundation (2021434953), and the Young Elite Scientist Sponsorship Program by CAST (YESS20210137). JY has received grants from the Academic Promotion Program of Shandong First Medical University (2019ZL002), Research Unit of Radiation Oncology, Chinese Academy of Medical Sciences (2019RU071), the foundation of National Natural Science Foundation of China (81627901, 81972863 and 82030082), the foundation of Natural Science Foundation of Shandong (ZR201911040452).

## Conflict of Interest

The authors declare that the research was conducted in the absence of any commercial or financial relationships that could be construed as a potential conflict of interest.

## Publisher’s Note

All claims expressed in this article are solely those of the authors and do not necessarily represent those of their affiliated organizations, or those of the publisher, the editors and the reviewers. Any product that may be evaluated in this article, or claim that may be made by its manufacturer, is not guaranteed or endorsed by the publisher.

## Supplementary Material

Supplementary Figure Kaplan-Meier curves for CSS and OS. Cancer-specific survival **(A)** and overall survival difference **(B)** in the three treatment groups. P<0.001 for CSS, P<0.001 for OS.

## Abbreviations

SEER, Surveillance, Epidemiology, and End Results; IO, immunotherapy; NSCLC, non-small cell lung cancer; CSS, cancer-specific survival; OS, overall survival; CRT, chemo-radiotherapy; PFS, progression-free survival; AC, adenocarcinoma.
