# Supplementary figures and images for: Treatment Patterns for Patients With Unresected Stage III NSCLC: Analysis of the Surveillance, Epidemiology, and End Results (SEER) Database
Source: Front Oncol. 2022 Jun 17;12:874022. doi: 10.3389/fonc.2022.874022 (PMC9248867; doi:10.3389/fonc.2022.874022)

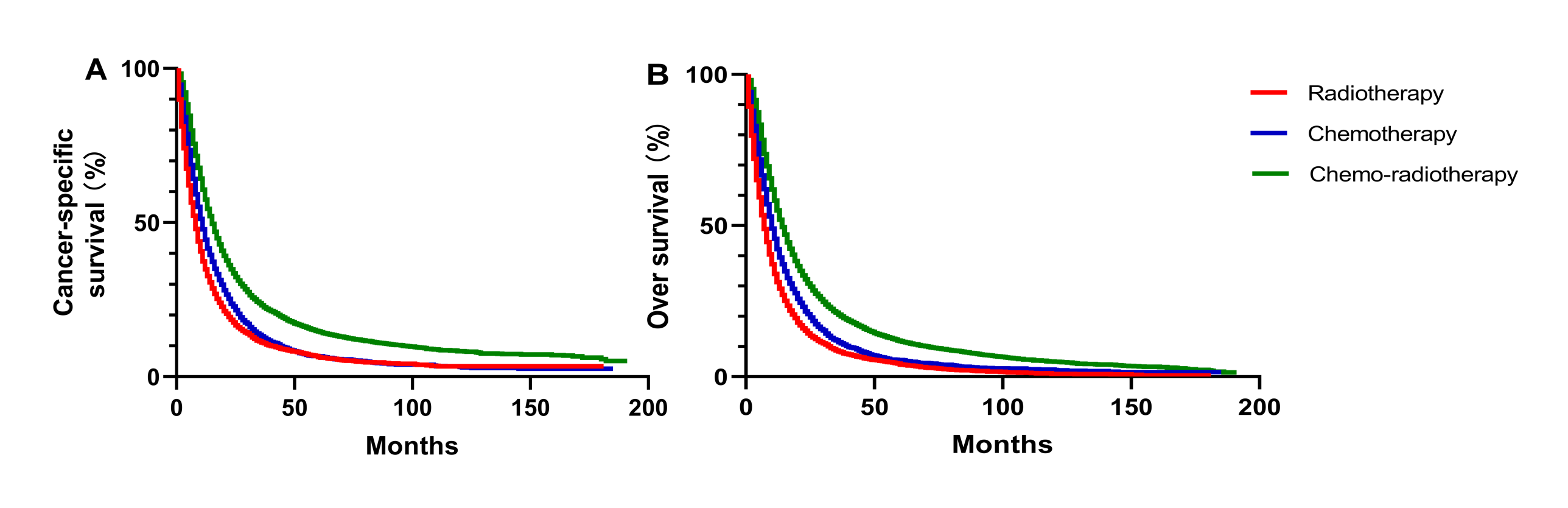

Supplement: Supplementary Figure — Kaplan-Meier curves for CSS and OS. Cancer-specific survival (A) and overall survival difference (B) in the three treatment groups. P<0.001 for CSS, P<0.001 for OS. [file Image_1.tif]
